# Do Immigrants Use Less Health Care than Non-immigrants? A Population-based Study among People living with Multimorbidity in Canada

**DOI:** 10.1093/eurpub/ckac129.505

**Published:** 2022-10-25

**Authors:** C Talukder, P Wilk, S Ali, S Stranges

**Affiliations:** 1 Department of Epidemiology and Biostatistics, Western University, London, Canada; 2 Institute of Social and Preventive Medicine, University of Bern, Bern, Switzerland; 3 Department of Precision Health, Luxembourg Institute of Health, Strassen, Luxembourg

## Abstract

**Background:**

Immigrants face unique health care barriers, which can negatively impact their health service use and overall health. Those with multimorbidity may face a particular challenge given its association with increased need for health care. The purpose of this study was to compare health care utilization, as measured by the number of visits to family physicians and specialists, between immigrants and Canadian-born individuals living with multimorbidity.

**Methods:**

A cross-sectional analysis was carried out using data from the 2015-2016 cycles of Canadian Community Health Survey (CCHS) on 9,014 study participants living with multimorbidity. The study utilized Andersen and Newman's behavioral model as a conceptual framework to identify quantifiable predictors associated with health service utilization. For the entire sample as well as for male and female subsamples, statistical models were fitted using negative binomial regressions to account for the count nature of the outcome variables.

**Results:**

After adjusting for relevant confounders, no statistically significant differences were observed between immigrants and Canadian-born respondents in the number of visits to family physicians or specialists. However, subgroup analysis revealed that female immigrants with multimorbidity had considerably fewer visits to family physicians than Canadian-born females (Incident Rate Ratio [IRR]=0.86, 95% CI: 0.76-0.98), while for males these differences were not significant (IRR=1.03, 95% CI: 0.87-1.21).

**Conclusions:**

Future research should focus on longitudinal studies to track the health status of immigrants over time, particularly those living with multimorbidity. Moreover, public health policies should be implemented to reduce cultural and social barriers to health care, with a special focus on female immigrants.

